# Fabrication of a Novel Nanoporous FeSiB Powder Catalyst via Annealing–Dealloying Synergistic Strategy for Enhanced p-Nitrophenol Degradation

**DOI:** 10.3390/ma19030629

**Published:** 2026-02-06

**Authors:** Qihang Yu, Ke Liu, Zhendong Sha

**Affiliations:** 1 State Key Laboratory for Strength and Vibration of Mechanical Structures, School of Aerospace Engineering, Xi’an Jiaotong University, Xi’an 710049, China; yuqihang@stu.xjtu.edu.cn; 2 Laboratory for Novel Disorder Materials, Xi’an Jiaotong University and Taixing Chengxing State Assets Management & Investment Co., Ltd., Xi’an 710049, China

**Keywords:** metallic glasses, chemical dealloying, nanoporous structure, degradation efficiency, catalysis

## Abstract

p-Nitrophenol (PNP), a highly toxic and recalcitrant organic pollutant prevalent in industrial wastewater, poses severe challenges to traditional Fenton treatment technologies. In this study, a novel nanoporous catalyst is synthesized via a combined annealing–dealloying strategy. Annealing at 550 °C and 600 °C induces partial crystallization, generating α-Fe and Fe_2_B phases that serve as preferential corrosion sites during chemical dealloying. This process results in a three-dimensionally interconnected nanoporous structure, which significantly increases the specific surface area of the catalyst to 2.642 m^2^/g. The optimized nanoporous catalyst exhibits excellent degradation performance, achieving complete removal of PNP within 30 min under room temperature reaction conditions. Notably, kinetic analysis reveals a degradation mechanism involving adsorption and Fenton-like catalysis. The high specific surface area provides abundant active sites for PNP adsorption, while the enhanced Fe^2+^ dissolution synergistically accelerates the degradation. The adsorption kinetic follows a pseudo-second-order model, and the degradation kinetic conforms to a first-order model, with activation energy analysis further confirming a surface-reaction-controlled process. This work provides a feasible approach and technical reference for designing efficient porous catalysts based on amorphous alloys for advanced treatment of refractory organic wastewater.

## 1. Introduction

p-Nitrophenol (PNP), a representative refractory phenolic organic pollutant commonly existing in wastewater from the chemical, pharmaceutical, and dye industries [1], exhibits high toxicity and poses potential carcinogenic, teratogenic, and mutagenic risks [2]. Its stable molecular structure is attributed to the strong electron-withdrawing effect of the nitro group (-NO_2_) on the benzene ring [3]. Consequently, PNP is resistant to microbial degradation in natural environments, making it a persistent hazardous substance in aquatic systems [4,5,6,7]. However, conventional wastewater treatment methods, including biological, physical, and chemical methods [8,9,10], are inefficient at degrading PNP contents to meet the requirements of the industrial wastewater discharge index due to the high stability of PNP molecules. Therefore, developing an efficient and cost-effective catalyst for the rapid removal of PNP has emerged as a significant challenge in environmental catalysis.

Advanced oxidation processes (AOPs) are regarded among the most promising technologies for addressing such recalcitrant organic pollutants [11,12,13]. By generating highly oxidative reactive species through physical, chemical, or photocatalytic ways, AOPs can non-selectively degrade large-molecule organic pollutants and further mineralize them into CO_2_, H_2_O, or small-molecule inorganic acids, thereby effectively avoiding secondary pollution [14,15]. However, the overall efficacy of AOPs heavily depends on the development of high-performance catalysts [16]. An ideal catalyst should possess high activity, exceptional stability, and abundant active sites.

Amorphous alloys, commonly known as metallic glasses (MGs), have a metastable alloy structure with short-range order but long-range atomic disorder due to the random arrangement of atoms [17]. Among them, Fe-based MGs, which can serve as unique zero-valent iron (ZVI) carriers, are regarded as highly effective catalysts for industrial wastewater treatment owing to their abundant exposure of surface-active sites [18,19]. Gas-atomized MG powders offer a larger specific surface area, which provides more active sites and improves catalytic efficiency [20,21]. The investigations conducted by Qin et al. [22] and Song et al. [23] confirmed the high efficiency and stability of the Fe-Si-B series amorphous powders in the degradation of dyes and nitrates. Nevertheless, the direct application of amorphous alloy powder as a catalyst encounters a fundamental limitation. Although its specific surface area exceeds that of bulk and strip materials, it remains inadequate when compared to porous materials, thereby restricting the complete exposure of active sites.

The porous materials prepared by dealloying can significantly increase the specific surface area of the catalyst, thereby enhancing its degradation performance [12,24,25,26,27,28]. Electrochemical dealloying enables precise control and produces more uniform structures, but its equipment is complex and more expensive [29,30]. In contrast, chemical dealloying offers less precise process control but is cost-effective and easy to operate, making it more suitable for industrial production [31,32]. Dealloying is classified into two types of mechanisms: composition-selective corrosion and phase-selective dissolution [33]. The electrode potential difference among various alloy components leads to the preferential corrosion of active components during the dealloying process, resulting in the formation of a porous structure. This holds significant implications for the field of catalysis. Wang et al. [34] prepared nanoporous copper powders by chemical dealloying, selectively leaching elements such as Zr and Al, and achieving efficient dye degradation across a wide pH range. Li et al. [35] used vapor-phase dealloying to produce hierarchical, bicontinuous nanoporous cobalt from a γ-CoZn alloy, creating a bimodal micro–nano porous structure via a two-step phase-transition process for potential catalytic and functional applications. However, single-component amorphous alloys, such as FeSiB, present challenges in forming a uniform and continuous porous structure through conventional dealloying, primarily due to the absence of an electrochemical potential difference in their homogeneous amorphous configuration, as noted in the research by Liu et al. [36]. This limitation constitutes a significant bottleneck that hinders the enhancement of their catalytic performance.

Heat treatment is commonly used to probe the thermal stability and structural heterogeneity of MGs by structural relaxation and subsequent crystallization [37]. Notably, nanocrystals form within the amorphous phase during heat treatment when the annealing temperature approaches the glass transition temperature, and their presence results in a potential difference within the alloy [38]. Therefore, applying heat treatment as a preprocessing step prior to dealloying is a critical strategy for generating porous structures in amorphous alloys.

In this study, we develop a nanoporous catalyst designed for the efficient degradation of p-nitrophenol (PNP). The objective is accomplished through an annealing–dealloying strategy in which heat treatment precedes dealloying. By precisely controlling the annealing and dealloying processes, uniformly distributed nanocrystalline phases are introduced into an amorphous matrix. Owing to their different chemical activities, these nanocrystals serve as sacrificial phases during selective corrosion in dealloying, resulting in a three-dimensional interconnected nanoporous structure with high specific surface area. To elucidate the degradation mechanism, adsorption kinetics and catalytic reaction kinetics are systematically applied to decouple and quantify the synergistic contributions of adsorption and catalysis. Supported by comprehensive material characterization, this kinetic insight shows that the remarkably enhanced performance results not only from the increased specific surface area for PNP enrichment but also from an accelerated surface-mediated catalytic reaction. Thus, our work provides an effective catalyst that addresses the technical challenge of forming porous structures from single-component amorphous alloys. We also provide a mechanistic understanding based on kinetic evidence, offering a rational pathway for designing high-performance porous catalysts for treating refractory organic wastewater.

## 2. Materials and Methods

### 2.1. Materials Preparation

Fe_83_Si_8_B_9_ metallic glassy powder prepared by gas-atomization technology was supplied by Hengbai Material Co., Ltd. (Nangong, China). p-Nitrophenol (PNP, 1 g/L) was obtained from Ruibiao Technology Co., Ltd. (Harbin, China). Sulfuric acid (H_2_SO_4_, 96–98 wt.%), granular sodium hydroxide (NaOH), hydrogen peroxide (H_2_O_2_, 27 wt.%), and alcohol (C_2_H_5_OH) were purchased from Sinopharm Chemical Reagent Co., Ltd. (Shanghai, China). All the chemical reagents used in this study were of analytical grade. Deionized water was used throughout the study.

### 2.2. Preparation and Characterization of Nanoporous Powder

The gas-atomized MG powder used for dealloying exhibits good spherical morphology with a maximum diameter of ~25 μm. To obtain a nanoporous structure, the MG powder was first annealed in a tube furnace under an argon atmosphere for 60 min at a heating rate of 20 K/min. Subsequently, the powder was rapidly quenched in water and then placed in a vacuum drying oven for 12 h. The resulting partially crystallized powder serves as the precursor material for chemical etching. The dealloying of the partially crystallized powder was conducted under free-corrosion conditions at room temperature in an aqueous sulfuric acid solution. After etching, the powder was rinsed at least three times with deionized water and anhydrous ethanol and immediately dried in a vacuum oven for 12 h.

The powder structure was analyzed by X-ray diffraction (XRD) with Cu Kα radiation. Microstructural morphology and elemental composition of the powder were characterized by scanning electron microscopy (SEM, Quanta 2000, FEI Company, Hillsboro, OR, USA) equipped with an energy-dispersive spectrometer (EDS). The surface chemical states of the dealloyed Fe-based powder were characterized by X-ray photoelectron spectroscopy (XPS) using a Kratos AXIS Ultra DLD instrument (Manchester, UK) with Al-Kα X-ray. The specific surface area of the powder was measured by the Brunauer–Emmett–Teller (BET, ASAP2020, Micromeritics, Norcross, GA, USA) method.

### 2.3. Degradation Experiment

The catalytic degradation of a 20 mg/L PNP solution was carried out in a 250 mL glass conical flask under water-bath heating. The PNP reaction solution was adjusted to an initial pH of 3.0 with 1 M H_2_SO_4_ or 1 M NaOH. In each experiment, 0.05 g/L of catalyst powder was used to activate 80 mM H_2_O_2_. The reaction temperature was maintained at 25 °C using a constant-temperature water bath, and the reaction was performed under mechanical stirring at 200 rpm. During the reaction, 5 mL aliquots were sampled at 10 min intervals, immediately quenched with 0.1 mL of 1 M NaOH, and analyzed by a UV–vis spectrometer (Lambda 35, PerkinElmer, Waltham, MA, USA) by monitoring the absorbance peak (λ_max_) at 400 nm [39]. Specifically, a series of PNP standard solutions with a concentration range of 0–20 mg/L was prepared. The absorbance was measured at pH = 9 and λ_max_ = 400 nm, yielding the following calibration curve equation: Abs = 0.063385C + 0.00699 (R^2^ = 0.9995), where Abs represents the absorbance, C represents the concentration, and R^2^ represents the fitting coefficient. All PNP concentration data reported in the manuscript were derived from this calibration curve, ensuring the accuracy of the quantitative results.

## 3. Results and Discussion

### 3.1. Effect of Annealing Temperature on the Porous Structure of Dealloying

**Figure 1:**
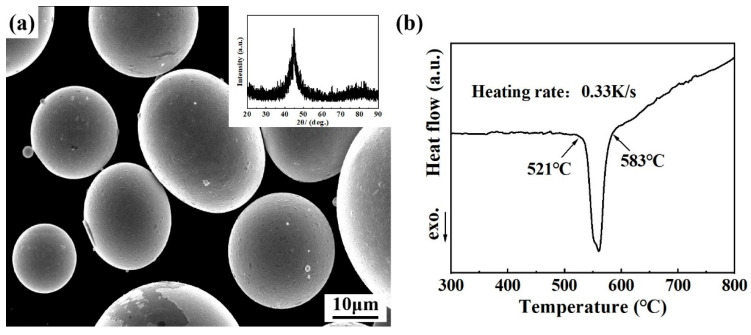
Characterization of the gas-atomized Fe_83_Si_8_B_9_ MGs powder: (**a**) SEM image (inset: corresponding XRD pattern); (**b**) DSC curve.

The microstructure and thermal properties of gas-atomized Fe_83_Si_8_B_9_ powder are presented in Figure 1. As shown in Figure 1a, the SEM image demonstrates that most particles have a favorable smooth spherical morphology. The inset figure in Figure 1a is the XRD pattern of the powder, which exhibits a broad diffraction peak at approximately 2θ = 45°, indicating a fully amorphous structure of the initial powder [40]. Figure 1b shows the differential scanning calorimetry (DSC) results of Fe_83_Si_8_B_9_ MGs powder, where the first crystallization temperature (T_x1_) is 521 °C, while the second crystallization temperature (T_x2_) is 583 °C. To obtain a nanocrystalline structure, Fe_83_Si_8_B_9_ MGs powder was heat-treated at temperatures of 500 °C, 550 °C, and 600 °C.

**Figure 2:**
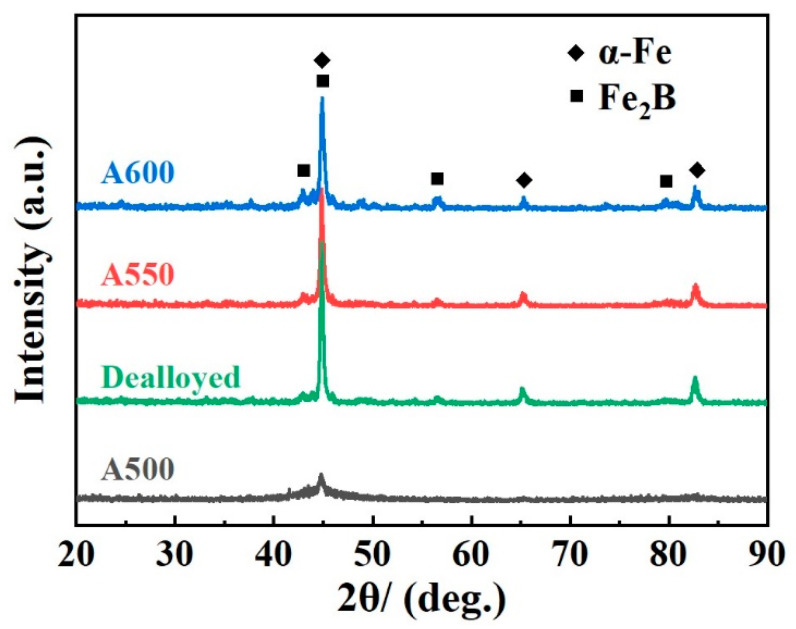
XRD patterns of the Fe_83_Si_8_B_9_ MGs powder after heat treatments at different temperatures and the powder after heat treatment at 500 °C after dealloying in 0.05 M H_2_SO_4_ solution for 20 min.

Figure 2 shows the XRD patterns of the Fe_83_Si_8_B_9_ MGs powder after heat treatments at different temperatures. Notably, the pattern of the powder annealed at 500 °C remains largely unchanged. In contrast, distinct diffraction peaks emerge after annealing at 550 °C and 600 °C. Peaks at 44.7°, 65.0°, and 82.3° correspond to the α-Fe phase, while those at 42.5°, 45.0°, and 79.5° are indexed to the Fe_2_B phase, confirming the crystallization of Fe_83_Si_8_B_9_ MGs powder. In addition, the amorphous feature still exists on the XRD pattern when the annealing temperatures are at 550 °C and 600 °C, indicating that Fe_83_Si_8_B_9_ MGs powder is partially crystallized. With increasing annealing temperature, peaks at approximately 56.0°, 65.0°, and 82.3° exhibit a slight increase, indicating an enhanced crystallization rate of the amorphous phase.

**Figure 3:**
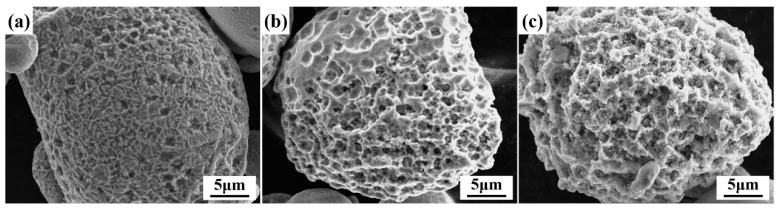
SEM images of the Fe_83_Si_8_B_9_ MG powder after annealing at different temperatures and dealloying in 0.1 mol/L H_2_SO_4_ solution for 30 min: (**a**) 500 °C, (**b**) 550 °C, (**c**) 600 °C.

Figure 3 shows the spherical morphology of the Fe_83_Si_8_B_9_ powder after annealing at different temperatures and dealloying in 0.1 mol/L H_2_SO_4_ solution for 30 min. After annealing at 500 °C (Figure 3a), the powder surface shows only mild etching without pore formation, which is attributed to the largely retained amorphous phase that provides enhanced corrosion resistance. In contrast, annealing at 550 °C and 600 °C (Figure 3b,c) leads to the development of a well-defined, three-dimensionally interconnected nanoporous structure. This transformation results from the formation of α-Fe and Fe_2_B crystalline phases above the crystallization temperature. Due to the lower corrosion potential of the α-Fe phase compared to the Fe_2_B phase, a local galvanic cell is established between the phases [41], promoting the selective dissolution of the α-Fe phase and thereby generating the nanoporous architecture. Based on these observations, the powders annealed at 550 °C and 600 °C were selected for further catalytic evaluation.

### 3.2. Effects of H_2_SO_4_ Concentration and Corrosion Time on the Degradation Performance

Based on the finding that annealing at 550 °C and 600 °C produces a three-dimensional interconnected porous structure on the spherical powder surface. This study investigates the effects of H_2_SO_4_ concentration and dealloying time on the PNP degradation performance of catalysts derived from MG powder annealed at these two temperatures. At 25 °C, 10 g/L MG powder was freely corroded in H_2_SO_4_ solutions of different concentrations (0.1 M, 0.2 M, and 0.3 M), with corrosion times of 10 min, 20 min, and 30 min.

**Figure 4:**
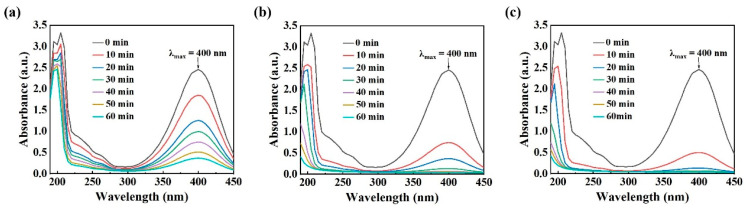
UV–visible absorption spectra of PNP by three types of powders after annealing at different temperatures and dealloying in 0.05 mol/L H_2_SO_4_ solution for 20 min: (**a**) original powder, (**b**) annealing at 550 °C, (**c**) annealing at 600 °C.

Figure 4 shows the UV–visible absorption spectra of PNP solutions treated with three types of powders. According to Lambert-Beer’s law, the solution concentration of PNP is proportional to its absorbance at the characteristic peak near 400 nm. Thus, the decrease in peak intensity directly reflects the extent of degradation. In Figure 4a, the peak intensity gradually declines over time, indicating steady but relatively slow removal of PNP. Figure 4b,c show the UV–visible absorption spectra of PNP by powders annealed at 550 °C and 600 °C, respectively. The characteristic absorption peak at 400 nm disappears after 40 min and 30 min, respectively, indicating near-complete PNP degradation. These results imply that the nitro bond (-NH4) in the PNP molecule is disrupted during the initial stage of degradation. Overall, the dealloyed Fe_83_Si_8_B_9_ MGs powder significantly accelerates PNP degradation, rapidly breaking the nitro bond and demonstrating excellent degradation performance.

The degradation of PNP by Fe (II)/Fe (III) oxides/hydroxides adheres to a first-order reaction model in chemical kinetics [42]. To quantitatively evaluate the influence of H_2_SO_4_ concentration and dealloying time on the degradation performance of PNP powder, the concentration data of the entire reaction period obtained during the experimental process were fitted by the first-order kinetics equation, as expressed in Equation (1):

(1) $$C_{t}/C_{0}=exp\left(-k_{obs}t\right)$$

where *k_obs_* is the reaction rate constant, *t* is the degradation time, *C*_0_ is the initial concentration of the solution, and *C_t_* is the concentration of the solution at time *t*.

**Figure 5:**
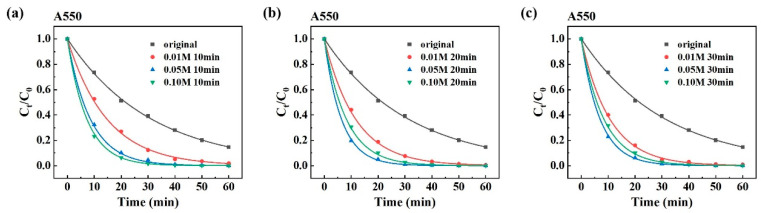
The influence of H_2_SO_4_ concentration and dealloying time on the degradation performance of the powder at 550 °C. (**a**) 10 min, (**b**) 20 min, (**c**) 30 min.

**Figure 6:**
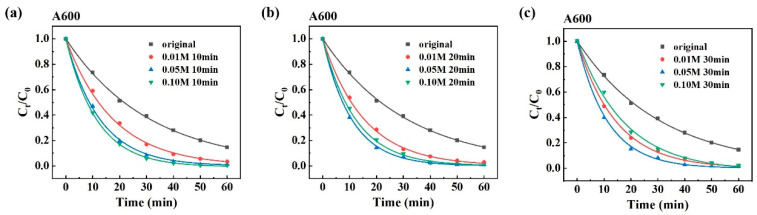
The influence of H_2_SO_4_ concentration and dealloying time on the degradation performance of the powder at 600 °C. (**a**) 10 min, (**b**) 20 min, (**c**) 30 min.

The influence of H_2_SO_4_ concentration and dealloying time on the degradation performance of the powder annealed at different temperatures is presented in Figure 5 and Figure 6, respectively. For clarity in subsequent discussion, the prepared powder is labeled as A550T10C0.01, indicating it is synthesized by annealing at 550 °C and dealloying in a 0.01 M H_2_SO_4_ solution for 10 min. As shown in Figure 5a, increasing sulfuric acid concentration during the dealloying process significantly enhances the degradation performance of the powder. This enhancement can be attributed to the rapid dissolution of the crystal structure, creating numerous small pores and increasing the specific surface area. However, when the dealloying time is extended to 20 and 30 min (Figure 5b,c), the powders A550T20C0.05 and A600T20C0.05 outperform A550T20C0.10 and A600T20C0.10, respectively. This suggests that excessively high acid concentration or prolonged etching may lead to pore coarsening and ligament refinement, ultimately reducing the overall porosity and accessible surface area.

To further elucidate the effects of sulfuric acid concentration and dealloying time on degradation performance, the reaction rate constant for PNP degradation over various samples is summarized in Table 1. The results indicate that the dealloyed powder annealed at 550 °C demonstrates significantly better degradation performance than that annealed at 600 °C, which is possibly attributed to the nucleation and growth theory. As the annealing temperature increases, nanograins tend to coarsen, and their number decreases. Annealing at 550 °C yields a finer and more uniform nanoporous ligament network, providing the highest specific surface area and the greatest number of active sites. In contrast, annealing at 600 °C may lead to excessive coarsening of α-Fe grains. During dealloying, the corrosion of these larger grains could result in oversized or poorly interconnected pores, which would reduce the accessible surface area and the number of active sites, thereby lowering catalytic performance [43]. Moreover, the influence of sulfuric acid concentration and dealloying time on degradation performance follows similar trends, as shown in Figure 5 and Figure 6. To gain deeper insight into how these factors affect the porous structure, the sample annealed at 550 °C was selected for an investigation into its morphological and structural evolution during dealloying in H_2_SO_4_ solution.

### 3.3. Material Characterization Analysis

**Figure 7:**
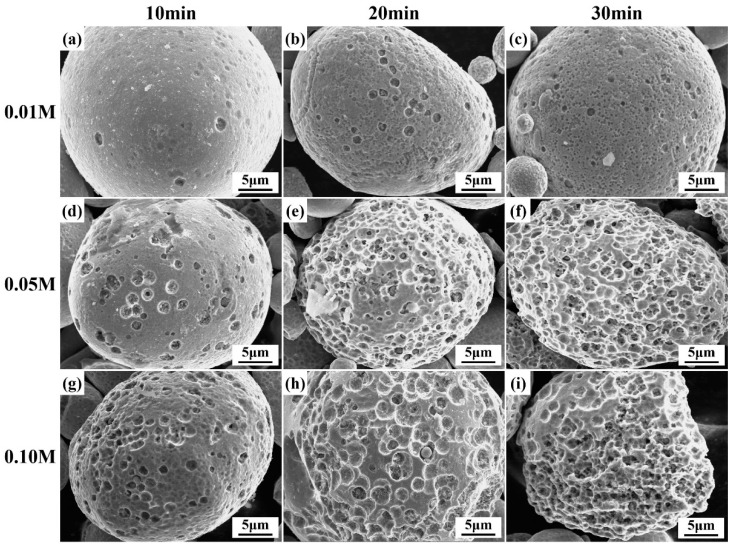
SEM of MGs powder annealed at 550 °C and dealloyed in different H_2_SO_4_ concentrations for different times: (**a**) 0.01 M 10 min, (**b**) 0.01 M 20 min, (**c**) 0.01 M 30 min, (**d**) 0.05 M 10 min, (**e**) 0.05 M 20 min, (**f**) 0.05 M 30 min, (**g**) 0.10 M 10 min, (**h**) 0.10 M 20 min, (**i**) 0.10 M 30 min.

Figure 7 shows the morphology of MG powder annealed at 550 °C and dealloyed in 0.01 M, 0.05 M, and 0.10 M H_2_SO_4_ solutions for durations of 10 min, 20 min, and 30 min, respectively. In the initial stages of dealloying, α-Fe grains preferentially dissolved in the 0.05 M and 0.10 M H_2_SO_4_ solutions. As etching proceeds, adjacent α-Fe grains dissolve successively, leading to the formation and interconnection of pores and eventually resulting in a nanoporous structure. Figure 7a–c show the morphology of the powder after dealloying in 0.01 M H_2_SO_4_ solution for 10 min, 20 min, and 30 min, respectively. The α-Fe grains on the spherical surface dissolve, producing pit structures, while the α-Fe grains in the interior remain intact. This limits the development of a three-dimensionally connected pore network. As shown in Figure 7d–i, increasing the H_2_SO_4_ concentration and extending the dealloying time produce progressively thinner ligaments and more pronounced surface pores, yielding a three-dimensional porous structure of interconnected pores and ligaments. This observation suggests that a low-concentration H_2_SO_4_ solution applied for a short time produces incomplete corrosion of α-Fe grains. However, when both acid concentration and etching time exceed a certain threshold, excessive corrosion dissolves much of the ligament material that connects the pores, causing the collapse of the nanopore network and the destruction of the pore architecture, as shown in Figure 7f,i. The results indicate that H_2_SO_4_ concentration significantly influences void formation, while dealloying time primarily affects void quantity. As H_2_SO_4_ concentration increases, the dissolution rate of α-Fe grains accelerates, producing a greater number of pores over time.

The atomic percentages of Fe, Si, B, and O for the samples annealed at 550 °C under different dealloying conditions are shown in Table 2. With the increase in acid concentration or etching time, the Fe content decreases significantly, while the O content increases substantially. In contrast, the contents of Si and B remain relatively stable. This indicates that the dealloying process selectively dissolves the α-Fe grains and promotes surface oxidation, which is consistent with the formation of the porous structure shown in Figure 7. Moreover, when the dealloying conditions are excessively harsh, the Fe content decreases significantly to 36.79 at.%. This indicates substantial loss of the iron matrix due to excessive corrosion. This finding corresponds well with the phenomena observed in Figure 7f,i, namely, the refinement of ligaments, destruction of the pore structure, and the partial collapse. These results demonstrate that excessive corrosion not only drastically reduces the Fe content but also compromises the integrity of the formed nanoporous structure, thereby negatively impacting the performance of the catalyst.

**Figure 8:**
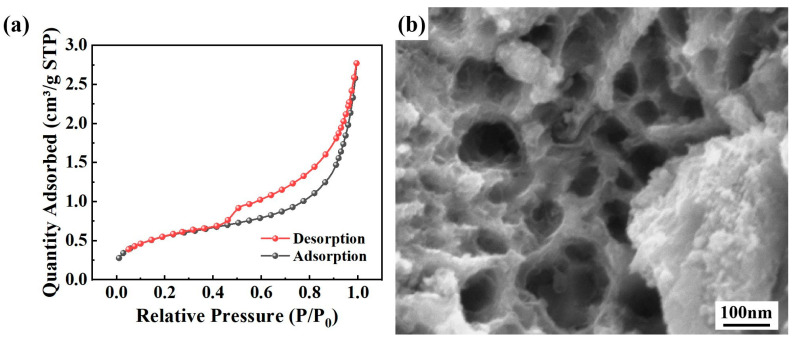
N_2_ physisorption analysis of the A550T20C0.05 powder: (**a**) N2 adsorption and desorption isotherms; (**b**) SEM of nanoporous structure.

BET measurement of the specific surface area of the dealloyed powders was conducted by the nitrogen (N_2_) adsorption–desorption technique. Figure 8 presents the obtained N_2_ adsorption–desorption isotherms and the SEM image of the pore size of the A550T20C0.05 powder. As shown in Figure 8a, the specific surface area of the A550T20C0.05 powder is 2.642 m^2^/g, which is nearly 10 times higher than that of the original powder (0.278 m^2^/g). Moreover, it is obvious that the N2 adsorption and desorption isotherms of Figure 8a contain a steep desorption step at around 0.5 p/p0. This is a typical characteristic of cavitation-induced desorption that is mainly attributed to the adsorption of mesoporous structure (i.e., pore size 2–50 nm) [44]. This is consistent with the SEM figure in Figure 8b. These results suggest that chemical dealloying effectively increases the specific surface area of the MG powder, thereby providing more accessible active sites and contributing to its enhanced degradation performance.

**Figure 9:**
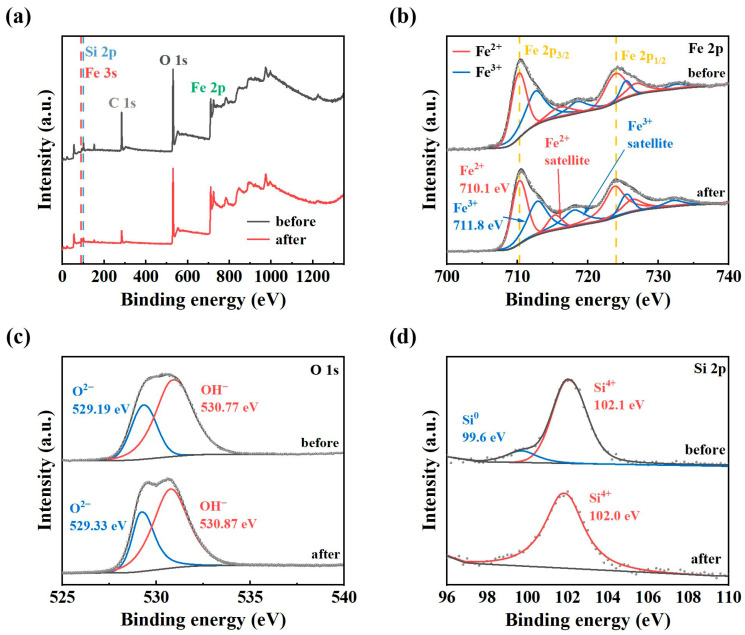
XPS spectra of A550T20C0.05 powder before and after degradation: (**a**) full spectrum, (**b**) Fe 2p, (**c**) O 1s, (**d**) Si 2p.

Figure 9a displays the XPS of A550T20C0.05 powder before and after the degradation. The detected elements on the powder surface include Fe 3p, Fe 2p, Si 2p, and O 1s. Detailed scanning is focused on the peaks of Fe 2p, O 1s, and Si 2p. The spectra for each element are shown in Figure 9b–d.

Figure 9b shows the Fe 2p spectra of the A550T20C0.05 powder before and after degradation. Before the reaction, the characteristic peaks at 710.7 eV and 712.8 eV correspond to Fe^2+^ (FeO) and Fe^3+^ (Fe_2_O_3_/FeOOH), respectively [45,46]. Notably, the absence of a peak around 707.2 eV indicates that no metallic Fe^0^ remains on the surface, confirming its oxidation during the dealloying process. Both before and after the degradation, the Fe 2p region can be deconvoluted into two chemical valence states: Fe^2+^ (711.28 eV) and Fe^3+^ (713.15 eV). Peak areas in the XPS spectra allow for qualitative analysis of the substance content, and the surface concentration of Fe^2+^ in A550T20C0.05 powder increases after degradation relative to its level before degradation.

Figure 9c presents the O 1s energy spectrum of A550T20C0.05 powder before degradation, which displays two chemical valence states: O^2−^ (529.19 eV) and OH^−^ (530.77 eV). After 60 min of degradation, the surface exhibits two characteristic peaks for O^2−^ and OH^−^ that are primarily associated with Fe-O (529.33 eV) and Fe-O/FeOOH (530.87 eV) chemical bonds [47]. Subsequently, the surface concentration of O^2−^ increases substantially, indicating the formation of additional Fe-O bonds during the reaction and a concomitant rise in Fe^2+^ concentration. This observation is consistent with the greater intensity of the Fe^2+^ characteristic peak after degradation than before.

As shown in Figure 9d, the Si 2p spectrum before degradation presents two valence stages. The peak at 102.1 eV(Si^4+^) is attributed to an iron silicate (Fe-Si-O) species, as its binding energy is significantly lower than that of SiO_2_ (103.4–103.8 eV) [46,47]. The signal at around 99.6 eV likely represents a combined contribution from the Fe 3s line and possibly a small amount of unoxidized silicon (Si^0^), which is attributed to peak overlap. After 60 min of degradation, the component at 102.1 eV remains stable, confirming the presence of a surface iron silicate layer. The feature at around 99.6 eV diminishes, suggesting surface changes during the reaction.

The above analysis indicates that after the dealloying treatment, the catalyst surface is fully oxidized, forming an active layer dominated by Fe (II)/Fe (III) oxides/hydroxides. Therefore, the efficient degradation of PNP does not originate from the homogeneous corrosion process of zero-valent iron (ZVI) but rather from a Fenton-like reaction catalyzed by the iron oxides on the nanoporous framework of the catalyst. The observed first-order kinetic model of this reaction derives from the enormous specific surface area provided by the nanoporous structure and the active layer dominated by Fe (II)/Fe (III) oxides/hydroxides.

The A550T20C0.05 catalyst prepared in this work demonstrates competitive degradation efficiency under ambient temperature and pH = 3 conditions. Its *k_obs_* value is significantly higher than those of many catalytic materials and comparable to some high-performance catalysts, as summarized in Table 3. Although certain noble-metal-based catalysts exhibit higher activity, the A550T20C0.05 catalyst shows promising cost-effectiveness and application potential due to its low-cost raw materials and simple preparation route.

### 3.4. The Mechanism of Degradation

The degradation of PNP by ZVI is characterized as a chemical reaction kinetic process that is activated by heat [53]. To determine the activation energy for the degradation reaction of the powder, the Arrhenius Equation (2) employed for calculation is as follows:

(2) $$lnk_{obs}=-E_{a}/RT+lnA$$

where *E_a_* is the activation energy, *R* is the gas constant with a value of 8.314 J·mol^−1^·K^−1^, *T* is the reaction temperature, and *A* is the preexponential factor.

**Figure 10:**
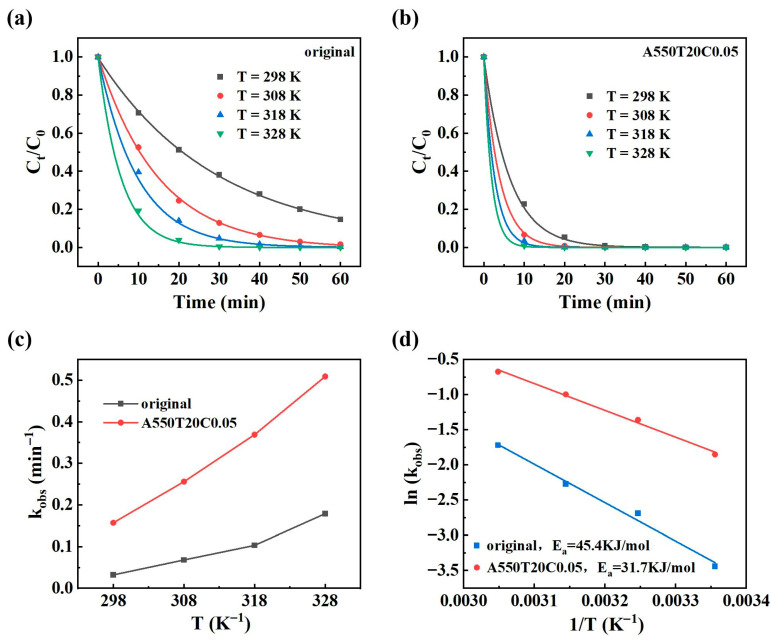
Effect of temperature on PNP degradation performance: (**a**) original powder, (**b**) A550T20C0.05 powder, (**c**) *k_obs_* values, (**d**) activation energies for reactions with the two powders.

Figure 10a,b show the PNP degradation performance of the original powder and A550T20C0.05 powder at different temperatures. The corresponding *k_obs_* values at different reaction temperatures are summarized in Figure 10c. The data indicate that *k_obs_* for both powders increase with reaction temperature, primarily due to the greater thermal motion of molecules, which accelerates the reaction rate. At the reaction temperature of 25 °C, the *k_obs_* values for the original powder and A550T20C0.05 powder were 0.032 min^−1^ and 0.157 min^−1^, respectively. At 55 °C *k_obs_* values increased to 0.168 min^−1^ and 0.509 min^−1^, respectively. Notably, the *k_obs_* of A550T20C0.05 powder at lower temperatures closely match that of the original powder at higher temperatures. These results suggest that A550T20C0.05 powder has a broader temperature adaptability range and retains high reactivity at lower temperatures compared with the original powder.

To further distinguish the control steps of the reaction, the activation energies for reactions with the two powders are shown in Figure 10d. The activation energies for PNP degradation are 45.4 kJ/mol for the original powder and 31.7 kJ/mol for the A550T20C0.05 powder. The degradation process of dye molecules occurs in two stages: the diffusion of molecules to the reaction site and the chemical reactions on the surface. Therefore, the PNP degradation may follow the same stage. Research indicates that the activation energies for diffusion-controlled reactions typically range between 8 and 20 kJ/mol, whereas those for chemically controlled reactions generally fall between 21 and 80 kJ/mol [16]. Consequently, both powders participate in degradation processes controlled by chemical reactions. Specifically, PNP molecules diffuse to reactive active sites, adsorb onto these sites, and undergo chemical reactions on the catalyst surface concurrently.

Given that adsorption is a critical precursor step in catalytic reactions, the adsorption capacity of three powders (A550T30C0.05, A550T20C0.10, and A550T20C0.05) for PNP was systematically assessed. The adsorption capacity of the catalyst is determined by Equation (3):

(3) $$Q_{t}=\left(C_{0}-C_{t}\right)\cdot V/m$$

where *Q_t_* represents the adsorption capacity of PNP, and *C*_0_ and *C_t_* represent the PNP concentration at an initial and arbitrary time, respectively. *V* represents the volume of the solution, and *m* represents the mass of the adsorbent.

**Figure 11:**
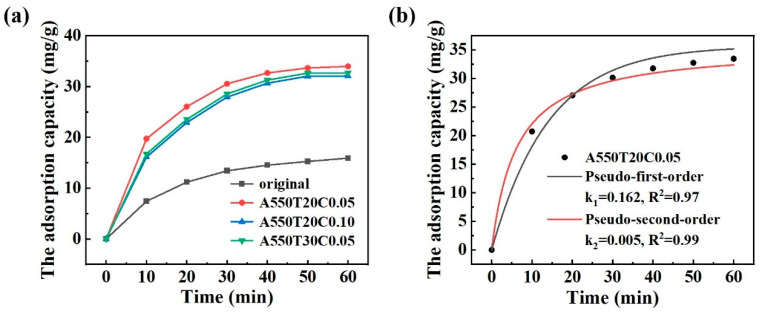
(**a**) Adsorption capacity of different powders and (**b**) adsorption kinetics of PNP on A550T20C0.05.

As shown in Figure 11a, adsorption equilibrium was achieved in all cases after 50 min. The original powder exhibits limited adsorption capacity for PNP, whereas the A550T30C0.05, A550T20C0.10, and A550T20C0.05 powders demonstrate superior adsorption performance. Notably, the A550T20C0.05 powder reaches the highest adsorption capacity of 35.69 mg/g for PNP. This enhancement can be attributed to two primary factors: first, the presence of numerous porous structures increases the specific surface area of the catalyst; second, the formation of oxide particles on the surface provides additional active sites.

For a deeper comprehension of the adsorption process, the obtained data were fitted to both the pseudo-first-order and pseudo-second-order models. As shown in Figure 11b, the pseudo-second-order model demonstrates a higher correlation coefficient R^2^ than the pseudo-first-order model, indicating that the PNP adsorption on the A550T20C0.05 powder follows the pseudo-second-order kinetics. This result suggests that the adsorption rate is governed by the number of active sites on the surface, and the adsorption process involves electron sharing or transfer between adsorbate and adsorbent, a characteristic of chemisorption [54].

The pseudo-first-order model is

(4) $$\log\left(Q_{e}-Q_{t}\right)=logQ_{e}-k_{1}t/2.303$$

The pseudo-second-order model is

(5) $$t/Q_{t}=1/k_{2}\cdot Q_{e}^{2}+t/Q_{e}$$

where *Q_e_* and *Q_t_* are the adsorption capacity at equilibrium and at any time *t*, respectively. *k*_1_ and *k*_2_ are the rate constants of the kinetic model.

To further investigate the degradation mechanism of PNP in the Fenton-like reaction, the impact of the adsorption performance of A550T20C0.05 powder on the catalytic efficiency during the oxidation process was examined. The degradation rate of PNP is determined using Equation (6):

(6) $$D=(C_{0}-C_{t})/C_{0}\times 100\%$$

where *D* is the degradation efficiency, *C*_0_ is the initial concentration of the solution, and *C_t_* is the concentration of the solution at time *t*.

**Figure 12:**
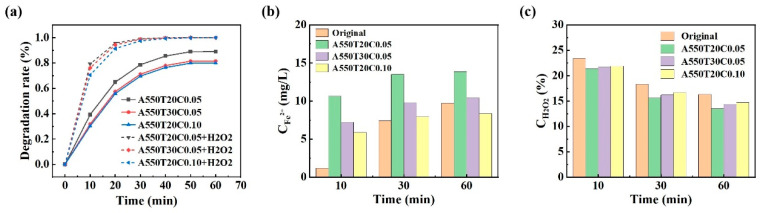
(**a**) Degradation of PNP under two conditions: with and without H_2_O_2_, (**b**) Fe^2+^ leaching rates for the four materials in 20 mg/L PNP solution at 25 °C, (**c**) H_2_O_2_ concentration during the Fenton-like reaction of four materials at 25 °C.

To elucidate the roles of adsorption and catalytic oxidation in the overall degradation, the PNP degradation efficiency under the catalyst alone and the catalyst with simultaneous H_2_O_2_ conditions is compared in Figure 12a. At 20 min, the degradation rates (adsorption) are about 72.1% for both the catalyst-only and catalyst/H_2_O_2_ groups. After 50 min, PNP degradation in the catalyst-only system reaches 80% and remains unchanged, indicating the adsorption capacity of the powder has saturated. In contrast, the catalyst/H_2_O_2_ system plateaued after 30 min, with PNP nearly completely degraded. These results indicate that strong adsorption enhances PNP removal during the oxidation process. Moreover, the degradation performance of the three powders consistently follows the order A550T20C0.05 > A550T30C0.05 > A550T20C0.10, independent of the presence of H_2_O_2_. This observation is discussed further below.

The classic Fenton process is based on Fe^2+^ in water reacting with H_2_O_2_ to generate •OH [55]. As a strong oxidizing radical, •OH attacks PNP molecules and cleaves the nitro bond, promoting degradation. The advanced Fenton process employs ZVI to produce Fe^2+^ under acidic conditions, thereby enhancing Fenton-like reactions. This reaction is shown in Equations (7) and (8):

(7) $$Surface-{Fe}^{0}+H_{2}O_{2}\to {Fe}^{2+}+2{OH}^{-}$$

(8) $${Fe}^{2+}+H_{2}O_{2}\to {Fe}^{3+}+{OH}^{-}+•OH$$

The Fenton-like process employed in this study exhibits an enhancing effect, as the nanoporous powder continuously leaches Fe^2+^. As shown in Figure 12b,c, to evaluate how the powders affect catalytic activity, the leaching rates of Fe^2+^ and the concentration of H_2_O_2_ in the solution of four powders were measured after 10 min, 30 min, and 60 min of reaction. The concentration Fe^2+^ in the reaction solution was determined using the 1,10-phenanthroline spectrophotometric method, and the residual concentration of H_2_O_2_ was measured via potassium permanganate titration.

Figure 12b presents the temporal change in Fe^2+^ leaching rates for the four materials in a 20 mg/L PNP solution at 25 °C. The data indicates that the leaching rates of Fe^2+^ for all materials increase over time and eventually stabilize with the prolonged reaction. Notably, the original powder consistently shows the lowest Fe^2+^ leaching rate throughout the reaction, and after 10 min, its leaching rate is markedly lower than those of the other three nanoporous materials. This indicates that the original powder possesses superior stability, while the nanoporous powder displays relatively high initial activity during the Fenton-like reaction process. Among the three porous materials, the A550T20C0.5 powder exhibited the highest Fe^2+^ leaching rate, which is attributed to excessive corrosion of the metallic iron matrix during the dealloying process. Figure 12c shows the temporal variation of H_2_O_2_ concentration during the Fenton-like reaction of the four materials at 25 °C. The concentration of H_2_O_2_ in the solution decreases over time for all materials, consistent with the consumption of H_2_O_2_ to produce •OH in a Fenton-like reaction. Notably, the post-reaction H_2_O_2_ concentration is the highest following treatment with the original powder. This observation, together with its low Fe^2+^ leaching rate, indicates that the original powder has the weakest capacity for H_2_O_2_ consumption and thus undergoes a relatively mild reaction. By contrast, the concentration of H_2_O_2_ in the solution of A550T20C0.05 powder is the lowest, consistent with its high Fe^2+^ leaching rate. This finding implies that A550T20C0.05 powder is relatively efficient at catalyzing the decomposition of H_2_O_2_, while the A550T20C0.10 and A550T30C0.05 powders show intermediate H_2_O_2_ consumption.

Based on the discussion above, the degradation mechanism of PNP in this study is primarily attributed to a synergistic combination of adsorption and Fenton-like catalysis. The formation of nanoporous structures notably increases the specific surface area of the powder, providing numerous active sites for the adsorption of PNP molecules. Moreover, dealloying markedly increases the initial activity of the material, while the elevated leaching rate of Fe^2+^ enhances the catalytic efficiency of H_2_O_2_.

**Figure 13:**
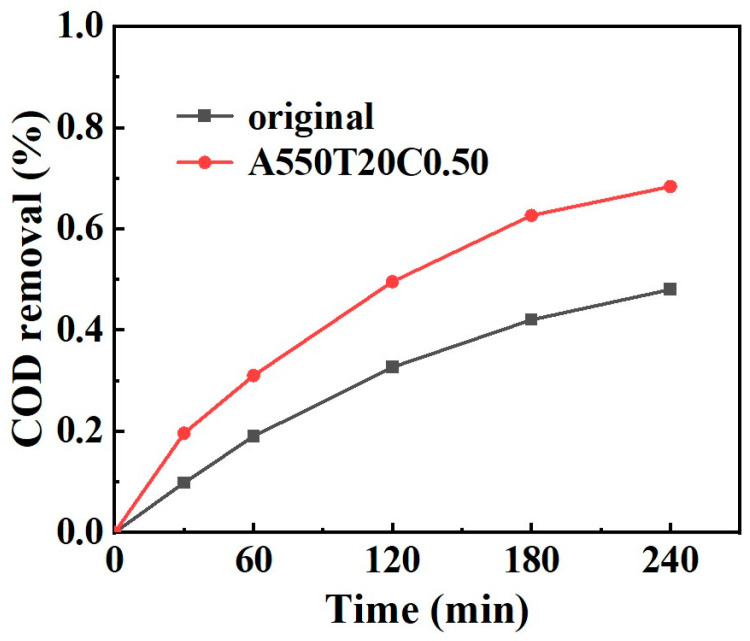
COD removal of original powder and A550T20C0.50 powder.

The above analysis confirms the high efficiency of the nanoporous catalyst in removing PNP. To further evaluate the thoroughness of degradation and the potential environmental safety of the treated wastewater, the removal rate of Chemical Oxygen Demand (COD) was investigated. As shown in Figure 13, after 30 min of treatment with the optimal catalyst A550T20C0.05, the COD removal reached approximately 19.6%, which is higher than that of the original powder (~9.8%). As the reaction proceeded to 240 min, the PNP solution treated with A550T20C0.05 powder achieved a COD removal rate as high as 68.3%, significantly surpassing that of the original powder (~48%). The substantial reduction in COD strongly indicates that not only are the PNP molecules efficiently decomposed but also that the organic intermediates, which mainly contain quinone compounds and small-molecule aliphatic carboxylic acids, are mineralized into inorganic carbon (e.g., CO_2_ and H_2_O) and inorganic salts [56]. The quinone compounds are mostly toxic, while the small-molecule aliphatic carboxylic acids are non-toxic, thereby greatly reducing the total organic load and toxicity of the solution.

## 4. Conclusions

In this work, a nanoporous Fe-based catalyst is prepared by an annealing–dealloying composite strategy, which exhibits excellent performance for the degradation of PNP. The main conclusions are summarized as follows:

1. Annealing the Fe_83_Si_8_B_9_ MGs powder above its crystallization temperature (550–600 °C) induces the formation of α-Fe and Fe_2_B phases within the amorphous matrix. These phases serve as preferential corrosion sites during the dealloying process and facilitate the efficient formation of a three-dimensionally interconnected nanoporous structure.
2. The morphological features and catalytic performance of porous catalysts depend highly on the dealloying conditions. After annealing at 550 °C, a nanoporous structure with uniform pores forms when dealloying in 0.05 M sulfuric acid for 20 min. Under these conditions, the PNP degradation reaches its optimal level, achieving a degradation rate of 99% at 30 min. The specific surface area of the powder is 2.642 m^2^/g, which is nearly 10 times higher than that of the original powder (0.278 m^2^/g). Deviation from these optimal parameters results in either insufficient matrix corrosion or structural collapse, which reduces degradation performance.
3. The degradation of PNP is primarily driven by combined adsorption and catalytic oxidation. The nanoporous structure facilitates rapid adsorption and concentration of PNP molecules at numerous active sites. Enhanced Fe^2+^ dissolution effectively activates H_2_O_2_ to produce •OH radicals that oxidize the adsorbed pollutants. This adsorption–catalysis synergism allows for rapid and near-complete degradation of PNP, highlighting its significant potential for treating refractory organic wastewater.

## Figures and Tables

**Table 1:** Reaction rate constant for PNP degradation over various samples.

Sample	k_obs_ (min^−1^)	Sample	k_obs_ (min^−1^)
A550T10C0.01	0.068	A600T10C0.01	0.057
A550T20C0.01	0.086	A600T20C0.01	0.065
A550T30C0.01	0.094	A600T30C0.01	0.069
A550T10C0.05	0.117	A600T10C0.05	0.079
A550T20C0.05	0.157	A600T20C0.05	0.093
A550T30C0.05	0.143	A600T30C0.05	0.088
A550T10C0.10	0.138	A600T10C0.10	0.079
A550T20C0.10	0.122	A600T20C0.10	0.085
A550T30C0.10	0.119	A600T30C0.10	0.063

**Table 2:** EDS analysis of samples.

Samples	Elements (at.%)			
	Fe	Si	B	O
A550	71.74	7.91	9.14	11.21
A550T10C0.01	59.88	7.83	9.01	23.28
A550T20C0.01	59.05	7.92	8.87	24.16
A550T30C0.01	57.37	7.84	8.81	25.98
A550T10C0.05	51.53	7.57	8.66	32.24
A550T20C0.05	42.56	7.46	8.69	41.29
A550T30C0.05	38.74	6.75	8.27	46.24
A550T10C0.10	44.55	7.28	8.59	39.58
A550T20C0.10	38.33	6.92	8.24	46.51
A550T30C0.10	36.79	6.53	8.01	48.67

**Table 3:** Comparison of k_obs_ for PNP degradation using various catalysts.

Catalyst	Type	Kobs	Initial Concentration	Catalyst Dosage
A550T20C0.05	Fenton-like	0.157/min	20 mg/L	0.5 g/L
Fe_78_Si_9_B_13_ [48]	Electro-Fenton	0.093/min	20 mg/L	/
Fe^2+^ [49]	Conventional Fenton	0.122/min	100 mg/L	0.01 g
Fe NPs [50]	Fenton-like	0.012/min	15 mg/L	0.25 g/L
3% Cu/g-C_3_N_4_ [51]	Photocatalytic	0.04/min	20 mg/L	1.0 g/L
AgNPs/ZSM-5 [52]	Reduction (NaBH_4_)	1.2/min	500 mg/L	/

## Data Availability

The original contributions presented in this study are included in the article. Further inquiries can be directed to the corresponding authors.
